# Upper Urinary System Changes After Radical Cystectomy and Bricker Urinary Diversion: A Retrospective Evaluation of Functional and Radiological Parameters

**DOI:** 10.3390/jcm15135163

**Published:** 2026-07-02

**Authors:** Alp Akyol, Kasim Emre Ergun, Mustafa Serdar Kalemci, Adnan Simsir, Baris Altay, Mahmut Kusbeci, Fuat Kizilay

**Affiliations:** 1 Department of Urology, Akhisar Mustafa Kirazoglu State Hospital, Manisa 45200, Türkiye; alp.akyol@saglik.gov.tr; 2 Department of Urology, Faculty of Medicine, Ege University, İzmir 35100, Türkiye; mustafa.serdar.kalemci@ege.edu.tr (M.S.K.); adnan.simsir@ege.edu.tr (A.S.); ahmet.baris.altay@ege.edu.tr (B.A.); fuat.kizilay@ege.edu.tr (F.K.); 3 Department of Radiology, Faculty of Medicine, Ege University, İzmir 35100, Türkiye; mahmut.kusbeci@ege.edu.tr

**Keywords:** hydronephrosis, ileal conduit, kidney volume, radical cystectomy, renal parenchymal thickness, upper urinary tract

## Abstract

**Background/Objectives**: Our objective is to evaluate the long-term effects of radical cystectomy and Bricker ileal conduit urinary diversion on upper urinary tract function and structure, with a specific focus on radiological and laboratory changes in renal function. **Methods**: A retrospective analysis was conducted of 120 patients who underwent radical cystectomy (RC) and Bricker ileal conduit urinary diversion between 2010 and 2024. Clinical, laboratory, and radiological data were assessed over a 24-month follow-up period. Renal parenchymal thickness, kidney volumes, and hydronephrosis grades were analyzed, and the relationship between interventions and renal outcomes was examined. **Results**: Postoperative follow-up showed a progressive decrease in renal parenchymal thickness and kidney volume, particularly in the left kidney. Hydronephrosis occurred in both renal units, with higher rates on the left side. Despite these changes, only a subset of patients required intervention, typically due to sepsis, acute renal failure, or pain. No significant deterioration in median estimated glomerular filtration rate values was observed; however, patients with interventions showed higher anteroposterior diameter/parenchymal thickness ratios, suggesting increased parenchymal damage. **Conclusions**: Following radical cystectomy and Bricker ileal conduit diversion, mild to moderate hydronephrosis is not uncommon and does not always necessitate intervention, especially in patients without clinical symptoms. Radiological parameters like renal parenchymal thickness and kidney volume may be valuable for monitoring renal deterioration. Careful follow-up and selective intervention are crucial in preserving renal function in this patient group.

## 1. Introduction

Bladder cancer is among the most prevalent malignancies of the urinary tract and represents a major global health burden. It ranks as the 10th most common cancer worldwide and is especially prominent in men, with a significantly higher incidence compared to women. Despite advances in diagnosis and treatment, muscle-invasive bladder cancer (MIBC) continues to carry a high risk of disease progression and mortality [1].

RC with urinary diversion remains the gold standard surgical treatment for patients with MIBC. While this procedure offers oncological control, it is associated with substantial physiological alterations, particularly affecting renal function over time [2]. As life expectancy increases, long-term surveillance of the upper urinary tract in this patient population has become increasingly critical, yet clinical guidelines for such monitoring remain imprecise. However, concerns regarding long-term effects on the upper urinary tract have gained increasing attention. In particular, progressive changes in renal function and imaging-based anatomical alterations—such as hydronephrosis, reduced parenchymal thickness, and renal volume (RV) loss—raise critical questions regarding follow-up strategies [3].

The exact pathophysiology of upper urinary tract deterioration following Bricker ileal conduit diversion is multifactorial. It typically involves a combination of chronic low-pressure vesico-ureteral reflux, subclinical ascending infections, and anastomotic stricture [2]. Ureteroenteric strictures, in particular, occur in up to 3–15% of patients and are predominantly driven by ureteral ischemia, tension at the anastomosis, or extensive periureteral dissection [4]. If these subtle morphological and functional changes are not detected early, chronic obstruction can lead to irreversible parenchymal atrophy and end-stage renal disease, underscoring the critical need for reliable, early radiological predictive markers.

This study investigates how the upper urinary system is affected in the long term after radical cystectomy and Bricker urinary diversion, based on objective radiological parameters. Furthermore, it explores whether every radiological alteration warrants intervention, whether thresholds can be proposed for action, and whether the anatomical trajectory of the left urinary tract predisposes it to greater impairment. By clarifying these issues, this study aims to contribute to evidence-based postoperative surveillance and decision-making.

## 2. Materials and Methods

### 2.1. Study Population

This retrospective, single-center study evaluated long-term upper urinary tract outcomes in patients who underwent radical cystectomy with Bricker ileal conduit urinary diversion between 2010 and 2024 at Ege University Hospital. The study was approved by the institutional ethics committee (approval number: 24-5T/3).

### 2.2. Inclusion and Exclusion Criteria

**Figure 1:**
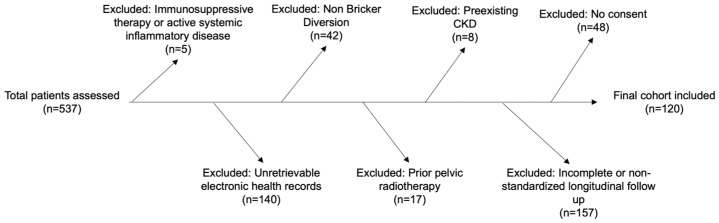
Flowchart of patient selection and exclusion criteria for final study cohort.

Out of 537 patients who underwent the surgery during the study period, a total of 120 patients were included based on specific inclusion and exclusion criteria. The substantial reduction from the initial cohort to the final study population was primarily driven by pragmatic, region-specific logistical challenges rather than early patient mortality or rapid oncologic progression. Our institution serves as a major tertiary referral center, attracting patients from vast geographic distances. In routine clinical practice within our healthcare system, patients residing in remote areas frequently miss or skip formal tertiary follow-up visits once they are asymptomatic. Furthermore, these patients often undergo surveillance at smaller local centers utilizing non-standardized equipment or alternative imaging modalities (such as ultrasonography or magnetic resonance imaging) that do not meet the strict, high-resolution longitudinal computed tomography protocol requirements of this study. Consequently, patients were excluded mainly due to these fragmented or non-compliant external imaging histories (*n* = 157) and incomplete 24-month CT records (*n* = 157), rather than selective attrition from adverse clinical outcomes. To evaluate potential survivorship bias, baseline clinical demographics (age, sex, and pathological T-stage) were compared between the included cohort (*n* = 120) and those excluded solely due to missing follow-up data (*n* = 157), revealing no statistically significant differences (*p* > 0.05). This empirical evidence is detailed in Supplementary Table S1. Inclusion criteria were availability of preoperative abdominal computed tomography (CT) imaging, laboratory, and pathology data, as well as postoperative follow-up CT scans and laboratory results at 6, 12, and 24 months. Informed consent for retrospective data review was also required. Patients were excluded from the initial 537 who were assessed, resulting in a final cohort of 120, primarily due to non-Bricker diversion, unavailability of required imaging or laboratory data, incomplete or non-standardized longitudinal 24-month follow-up, lack of consent, a history of pelvic radiotherapy, pre-existing chronic kidney disease, or use of immunosuppressive therapy/active systemic inflammatory disease (Figure 1).

### 2.3. Procedures and Evaluations

Patient data were obtained from hospital archives, electronic medical records, and phone interviews where applicable. All abdominal CT (Somatom Definiton Edge, Siemens Healthineers, Erlangen, Germany) scans were evaluated by a single experienced radiologist, with quantitative measurements performed separately for each kidney preoperatively and at 6, 12, and 24 months postoperatively. All radiological assessments were conducted separately for the right and left renal units. The following parameters were recorded bilaterally and independently preoperatively and at the 6th, 12th, and 24th postoperative months: renal pelvis anteroposterior (AP) diameter (mm), average renal parenchymal thickness (mm), renal volume, AP diameter/parenchymal thickness ratio, and hydronephrosis grade according to the Society for Fetal Urology (SFU) classification [5,6,7]. This side-specific approach enabled a detailed evaluation of unilateral changes in upper urinary tract morphology over time.

**Figure 2:**
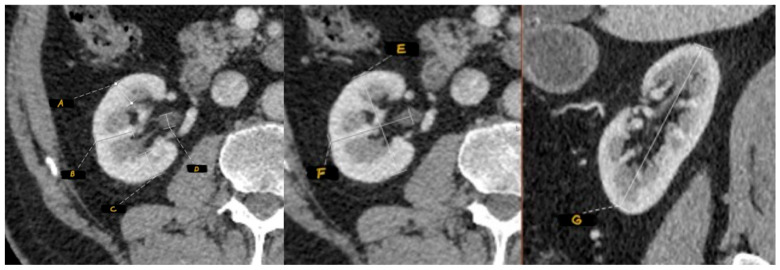
Measurements of the right renal pelvis AP diameter, average parenchymal thickness, and kidney volume on axial and sagittal CT imaging. A: Anterior parenchymal thickness (mm), B: lateral parenchymal thickness (mm), C: posterior parenchymal thickness (mm), D: renal pelvis AP diameter (mm), E: anteroposterior length of the kidney (mm), F: mediolateral width (mm), G: superoinferior height (mm).

Renal pelvis AP diameter was measured at the site where the pelvis exits the renal parenchyma. Parenchymal thickness was calculated by averaging three measurements (anterolateral, dorsolateral, and posteromedial) taken from axial CT slices at the level of the renal hilum. Renal volume was calculated using the ellipsoid formula: RV = width × depth × length × π/6 (Figure 2) [8].

In addition to imaging parameters, serum creatinine levels and eGFR values were also recorded at the corresponding follow-up time points to evaluate renal function. Hydronephrosis-related interventions, when necessary, were documented alongside radiological and clinical findings. All patients included in this study had previously undergone RC with Bricker ileal conduit urinary diversion due to muscle-invasive bladder cancer. Specifically, all procedures were performed exclusively by four senior surgeons from the authorship group (Adnan Simsir, Fuat Kizilay, Mustafa Serdar Kalemci, and Kasim Emre Ergun) who strictly adhered to an identical, standardized institutional protocol for open Bricker diversion and ureteroenteric anastomosis, thereby minimizing potential operator bias. The oncological part of the cystectomy was performed in accordance with established and widely accepted surgical principles described in the literature. In the urinary diversion phase, a 15 cm segment of ileum was isolated approximately 15 cm proximal to the ileocecal valve. Prior to division, the segment was assessed to ensure adequate mobility toward the abdominal wall without tension and to confirm preserved vascular supply, especially in obese patients, where longer conduit lengths were sometimes required. After verifying suitability, the ileal segment was transected using a non-crushing clamp and sharp dissection, with careful attention to mark the distal end intended for stoma creation. Continuity of the bowel was restored using a side-to-side ileoileal anastomosis with continuous sutures, and the mesenteric defect was closed with absorbable sutures [9]. Following this, both ureters were mobilized, with preservation of adventitial blood supply. The left ureter was transposed to the right side through a window carefully created in the sigmoid mesentery to avoid compression or ischemia. Ureteroenteric anastomosis was performed using the Bricker technique, in which the ureters were separately spatulated and anastomosed end-to-side to the proximal portion of the ileal conduit. Each anastomosis was constructed using interrupted full-thickness absorbable sutures (4-0 or 5-0 PDS or polyglactin) through both the ureter and ileal wall [4,9].

### 2.4. Statistical Analysis

Data analysis was performed using IBM SPSS Statistics for macOS (v29.0; IBM Corp., Armonk, NY, USA). Descriptive statistics were presented as frequency and percentage for categorical variables and as mean ± standard deviation or median (min–max) for continuous variables. Between-group comparisons were made using the Mann–Whitney U test, and categorical variables were compared using the Chi-square or Fisher’s exact test. Changes across multiple time points were analyzed using Friedman’s two-way analysis of variance by ranks. Logistic regression and receiver operating characteristic (ROC) curve analyses were additionally used to evaluate predictors of hydronephrosis intervention and to determine the discriminatory performance and optimal cutoff values of relevant imaging parameters. To adjust for potential over-optimism and ensure model robustness, given the relatively small number of outcome events (15 interventions), internal validation was performed for the primary predictor (12-month left AP diameter-to-parenchymal thickness ratio) using bootstrap resampling with 1000 replications. This allowed for calculation of an average model optimism and an optimism-corrected area under the curve (AUC). A *p*-value < 0.05 was considered statistically significant.

## 3. Results

A total of 120 patients were evaluated in the study. Since radiological measurements were taken separately for each kidney, 240 renal units were evaluated. Table 1 provides a comprehensive overview of their baseline characteristics, clinical findings, laboratory values, pathological features, and treatment details.

Hydronephrosis requiring intervention was observed in 12.5% (*n* = 15) of patients. Among these, 46.7% underwent antegrade double J stenting, and 53.3% required percutaneous nephrostomy. Acute kidney injury was the leading indication for intervention (46.7%), followed by renal colic pain (20%), tumor-related compression (20%), and ureterolithiasis (13.3%).

The median creatinine level increased from 0.9 mg/dL preoperatively to 1.1 mg/dL at 24 months, while the eGFR remained stable at a median of 60 mL/min/1.73 m^2^ throughout follow-up. Patients who underwent intervention had significantly higher creatinine levels at all time points (*p* < 0.05) and lower eGFR at baseline, 6 months, and 12 months compared to those without intervention.

**Figure 3:**
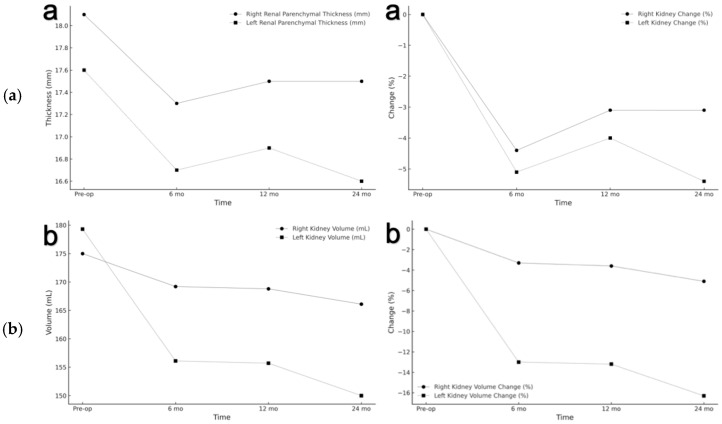
Time-dependent changes in renal parameters after radical cystectomy: (**a**) absolute and relative percentage changes in right and left renal parenchymal thickness; (**b**) absolute and relative percentage changes in kidney volumes.

Quantitative analysis of longitudinal changes demonstrated a progressive reduction in renal parenchymal thickness and volume over the 24-month follow-up period (Table 2). The median parenchymal thickness of the right kidney decreased by 3.3% at 6 months and remained relatively stable thereafter, whereas the left kidney exhibited a more pronounced reduction, reaching 5.7% by month 24 (Figure 3a). In terms of renal volume, the right kidney demonstrated a cumulative reduction of 5.3% at 24 months, while the left kidney volume declined more significantly, with a total decrease of 16.3% (Figure 3b). These findings suggest an asymmetric pattern of renal deterioration following Bricker ileal conduit diversion, with the left kidney being more affected over time.

Hydronephrosis severity (SFU grading) showed no major progression in most patients. At 24 months, Grade 0–1 hydronephrosis was observed in 96.9% of right kidneys and 91.7% of left kidneys. Only 2.1% of right kidneys and 6.3% of left kidneys developed Grade 4 hydronephrosis.

Significant differences in renal parameters were observed between patients with and without hydronephrosis interventions. At the 12-month mark, the right renal pelvis AP diameter and the left kidney AP diameter to parenchymal thickness ratio were significantly higher in the intervention group (*p* = 0.008 and *p* = 0.039, respectively).

**Figure 4:**
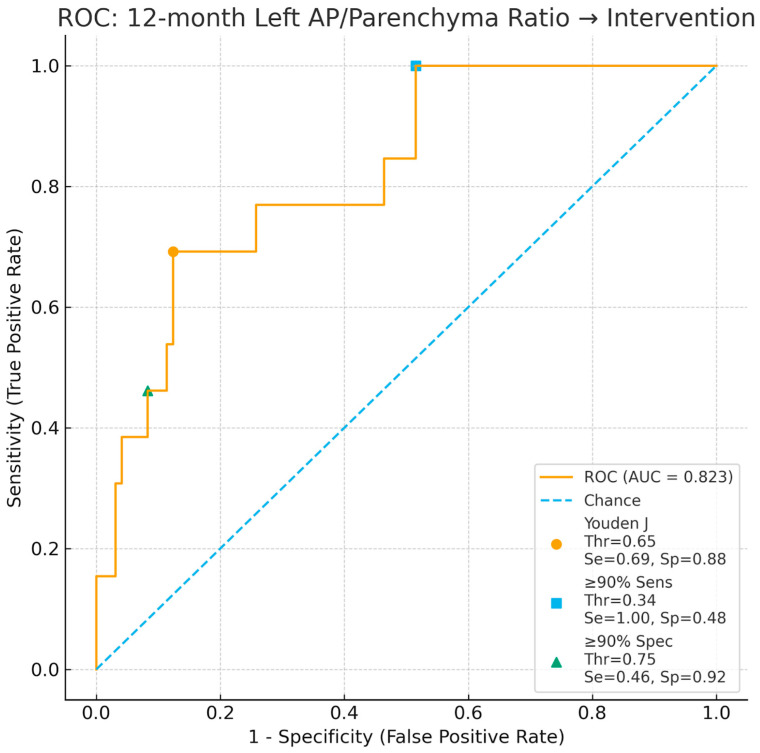
Receiver operating characteristic (ROC) curve demonstrating the discriminatory performance of the 12-month left renal pelvis AP diameter to parenchymal thickness ratio for predicting hydronephrosis intervention, with labeled thresholds corresponding to the Youden’s J optimal cutoff, ≥90% sensitivity, and ≥90% specificity.

To further address renal functional changes and identify independent predictors of hydronephrosis intervention, serial renal function parameters and multivariable logistic regression analyses were performed. Potential multicollinearity among the longitudinal measurements and primary confounders (diabetes mellitus, hypertension) was rigorously evaluated using variance inflation factors (VIFs). All included variables demonstrated highly acceptable VIF values ranging between 1.00 and 1.12 (VIF < 3), indicating no significant multicollinearity. In the multivariable model incorporating bilateral AP diameter to parenchymal thickness ratios across preoperative and 6-, 12-, and 24-month time points, the 12-month left renal ratio emerged as a significant independent predictor of intervention (95% CI 1.74–13.50, adjusted OR = 4.84, *p* = 0.003), while the 12-month right renal ratio and the preoperative left ratio demonstrated additional independent associations (*p* < 0.05). ROC curve analysis demonstrated a promising apparent discriminatory performance of the 12-month left ratio (AUC 0.82). To account for potential overfitting due to the limited number of events, internal validation was performed via bootstrap resampling (B = 1000). The average model optimism was estimated to be 0.018, yielding an optimism-corrected AUC of 0.805. While these internally validated findings suggest a potential exploratory value, the small event size necessitates a cautious clinical interpretation. An optimal cutoff of 0.65 yielded 69% sensitivity and 88% specificity for predicting the need for intervention (Figure 4). These findings validate that higher AP diameter to parenchymal thickness ratios, particularly at 12 months, are independently associated with subsequent hydronephrosis intervention and justify their use as predictive markers in this population.

When stratified by tumor stage, patients with T3–T4 disease exhibited significantly lower parenchymal thickness and renal volume in both kidneys at all time points compared to those with T0–T2 disease (*p* < 0.05). Similar reductions were observed in patients with lymph node metastasis and those who received adjuvant or neoadjuvant chemotherapy.

## 4. Discussion

This study aimed to evaluate the long-term impact of RC and Bricker ileal conduit urinary diversion on the upper urinary tract using objective radiological parameters. The most striking findings were the measurable decrease in renal parenchymal thickness and renal volume in both kidneys over a 24-month follow-up period, with significantly more deterioration observed on the left side. Moreover, while hydronephrosis was relatively common during follow-up, it did not universally necessitate intervention. Notably, patients who required intervention had significantly higher renal pelvic AP diameter to parenchymal thickness ratios, suggesting that this index may hold clinical value in identifying cases requiring active treatment.

A point worthy of clinical discussion is our utilization of the SFU grading system. Although universally recognized and highly reproducible for cross-study comparisons, the SFU system was inherently developed for pediatric and fetal populations. In adult patients who have undergone radical cystectomy and Bricker diversion, chronic non-obstructive upper tract dilatation due to altered reservoir compliance or low-pressure reflux is common. This physiological adaptation can reduce the clinical specificity of SFU grading when trying to pinpoint true mechanical obstruction. It is precisely this clinical limitation that drove the core objective of our study: because static visual grading is often insufficient in adults, we focused on quantitative, continuous geometric measurements, specifically the AP diameter-to-parenchymal thickness ratio, to directly integrate physical tissue loss into the clinical equation.

From a methodological standpoint, the retrospective design of this study limited the ability to assess certain causal relationships. Nonetheless, its strength lies in the objective and quantitative evaluation of renal changes based on serial CT measurements, including renal volume, parenchymal thickness, and hydronephrosis grading. The bilateral analysis of renal units and incorporation of percent changes over time further strengthened the ability to detect subtle yet clinically meaningful renal deterioration.

Clinically, these findings emphasize that upper urinary tract deterioration post-RC is not uniform and may be significantly influenced by anatomical factors related to surgical technique, especially concerning the left ureter. The requirement for greater dissection and transposition of the left ureter under the sigmoid mesentery likely contributes to the higher incidence of hydronephrosis and more pronounced renal deterioration on that side. Furthermore, our findings suggest that radiological findings alone may not always predict clinical outcomes, as not all patients with hydronephrosis required intervention, and many maintained adequate renal function during follow-up.

Yang et al. evaluated 249 patients who underwent either rectosigmoid (RS) pouch or ileal conduit diversion and reported a progression in mean serum creatinine levels from 1.02 mg/dL to 1.18 mg/dL over a 5-year follow-up period [10]. Similarly, in our cohort, the median preoperative serum creatinine level was 0.9 mg/dL, which increased to 1.1 mg/dL at the 24-month postoperative follow-up. Regarding eGFR, although no statistically significant change was observed in median eGFR values during follow-up, a notable decline was detected in minimum eGFR values. This finding suggests that, while most patients maintained stable renal function, those who exhibited deterioration experienced a progressive decline. In line with our observations, Fujiwara et al. investigated early and intermediate renal function outcomes following RC with ileal conduit diversion and found that mean eGFR values decreased from 69.6 mL/min/1.73 m^2^ at baseline to 60.8 mL/min/1.73 m^2^ at 1 year postoperatively [11]. Taken together, these results indicate that, although overall renal function remains stable in the majority of patients after radical cystectomy and ileal conduit urinary diversion, a subset of patients is susceptible to progressive renal impairment, emphasizing the need for individualized renal monitoring strategies.

Another key implication is the potential value of using specific radiological indices such as the renal pelvis AP diameter-to-parenchymal thickness ratio. This parameter showed consistent association with the need for intervention and may serve as an early indicator of clinically significant hydronephrosis, even in patients with otherwise stable serum creatinine or eGFR values. To our knowledge, no previous work has applied multivariable logistic regression to systematically evaluate radiographic predictors of clinically significant upper-tract obstruction in patients undergoing Bricker ileal conduit diversion. By integrating longitudinal CT-based measurements with objective clinical outcomes, the present study demonstrates that the renal pelvis AP diameter to parenchymal thickness ratio, particularly at 12 months, functions as an independent determinant of whether a renal unit ultimately requires intervention. This distinction is clinically important, as postoperative hydronephrosis after ileal conduit diversion is common but does not, in itself, mandate treatment unless accompanied by acute kidney injury, flank pain, or stone-related obstruction. Our findings therefore support the development of CT-based decision thresholds that can help differentiate benign postoperative dilatation from obstruction warranting decompression. The identification of a ratio threshold with high discriminatory accuracy provides a practical radiologic parameter that may guide surveillance strategies and refine intervention criteria in this patient population.

The findings also offer a nuanced view regarding the impact of perioperative chemotherapy. Patients receiving neoadjuvant or adjuvant chemotherapy experienced more pronounced radiological changes, particularly in renal pelvic dilation and parenchymal thinning, suggesting a potential additive nephrotoxic effect. These data underline the importance of close upper tract monitoring in patients receiving systemic therapies, especially when combined with anatomical factors that may compromise ureteral drainage.

Given that our study period spanned over a decade (2010–2024), potential temporal variations in clinical practice must be considered. However, all surgeries were performed at a single high volume tertiary academic center by senior staff urologists adhering to standard institutional protocols. Crucially, the core Bricker technique, the routine placement of standard 6Fr ureteral stents, and the postoperative stent retention period (21 days) remained homogenous throughout the study period. While the utilization of neoadjuvant chemotherapy evolved over the decade in accordance with guideline updates, this shift was explicitly analyzed as a distinct clinical variable. By stratifying for chemotherapy exposure, we were able to isolate its specific impact independent of the surgical methodology.

This study has several limitations that warrant careful consideration. First, its retrospective, single-center design restricts broad generalizability. Second, our strict selection criteria led to substantial attrition of the initial surgical population (from 537 down to 120 patients), introducing unavoidable selection and survivorship biases. Patients who dropped out of systematic imaging follow-ups may clinically represent a high-risk group involving early oncologic recurrence, severe complications, or death. Consequently, our final analyzed cohort likely reflects a healthier, more stable subpopulation, which may understate the true incidence and scale of long-term upper urinary tract deterioration. Furthermore, the baseline exclusion of prior CKD biases our long-term functional survival data toward more optimistic outcomes. Third, although we adjusted for major comorbidities (diabetes, hypertension) and clear clinical events (sepsis, acute kidney injury), retrospective models cannot fully account for unmeasured confounders, such as subclinical recurrent urinary tract infections, patient hydration habits, or mild metabolic acidosis fluctuations. Finally, our follow-up period was limited to 24 months. While a 2-year timeline is robust for identifying early-to-intermediate structural shifts and early failure patterns, it is too short to capture late-onset benign strictures or chart lifetime renal functional trajectories, which typically require 5 to 10 years of longitudinal tracking. Renal function assessment relied on serum creatinine and eGFR without functional imaging, potentially limiting the evaluation of segmental renal function.

Moving forward, the clinical application of the proposed AP diameter to parenchymal thickness ratio could be significantly enhanced by the integration of artificial intelligence (AI) and automated 3D radiomic segmentations. While manual CT measurements and the ellipsoid volume formula provide practical clinic-based estimations, AI-driven automated volumetric assessments could eliminate inter-reader variability and offer more precise real-time surveillance of parenchymal loss. Future prospective, multi-center studies should not only externally validate our 0.65 threshold, but also incorporate functional dynamic imaging (e.g., MAG3 renography) and novel serum renal biomarkers alongside these structural geometric indices. Such multimodal approaches will ultimately facilitate the development of personalized, risk-stratified follow-up algorithms for patients undergoing radical cystectomy.

## 5. Conclusions

This study shows that radical cystectomy with Bricker ileal conduit impacts upper urinary tract anatomy and renal function over time. Radiological findings indicate a progressive decline in parenchymal thickness and kidney volume, especially in the left kidney, suggesting technical vulnerability. Although overall renal function remained stable at the cohort level, the combined radiological and biochemical findings suggest that structural alterations may emerge earlier than measurable declines in serum markers for a subset of vulnerable patients. The preferential loss of left-sided parenchymal tissue and the elevated AP diameter to parenchymal thickness ratios among units requiring intervention indicate that CT-derived anatomical parameters can reveal early signals of developing obstruction or parenchymal compromise. However, due to our small event size (15 interventions), these thresholds must be interpreted strictly as preliminary, exploratory, and hypothesis-generating observations. They do not constitute validated clinical decision-making algorithms at this stage. Extensive external validation within larger, prospective, independent, multicenter cohorts is mandatory before the AP diameter to parenchymal thickness ratio can be recommended for daily clinical implementation. Despite the inherent radiation exposure, such quantitative imaging metrics provide a level of localization and sensitivity that traditional renal function tests cannot offer, supporting their role as adjunctive tools in monitoring high-risk renal units following Bricker ileal conduit diversion. Both neoadjuvant and adjuvant chemotherapy are linked to renal deterioration. These results highlight the importance of proper patient selection, careful ureteral handling, and structured radiological follow-up to maintain long-term renal function.

## Figures and Tables

**Table 1:** Demographic, clinical, laboratory, pathological, and treatment-related characteristics of the study population (*n* = 120).

Variables * (*n* = 120)	*n* (%)	Mean ± SD	Median (Min–Max)
Age		63 ± 9	64 (27–80)
Sex			
Male	100 (83.3)		
Female	20 (16.7)		
Smoking	101 (84.2)		
Coronary artery disease	33 (27.5)		
Diabetes mellitus	36 (30)		
Hypertension	46 (38.3)		
Exitus	54 (45)		
Overall survival (Months)		53.78 ± 40.81	37 (6–168)
Preoperative creatinine (mg/dL)		1.08 ± 0.4	0.9 (0.57–2.4)
Creatinine at hydronephrosis intervention (mg/dL)		3.01 ± 2.17	2.2 (0.79–8)
Time to intervention for hydronephrosis (months)		15.27 ± 8.95	13 (6–36)
Lymph node metastasis	33 (27.7)		
Positive surgical margin	14 (11.8)		
Chronic hemodialysis	5 (4.2)		
Neoadjuvant chemotherapy	30 (25.0)		
Adjuvant chemotherapy	33 (27.5)		
Pathological T stage (*n* = 118)			
CIS	2 (1.7)		
T0	1 (0.8)		
T1	12 (10.2)		
T2	52 (44.1)		
T3	38 (32.2)		
T4	10 (8.5)		
Ta	2 (1.7)		
Tis	1 (0.8)		
Histological subtype (*n* = 119)			
Adenocarcinoma	1 (0.8)		
Epithelial malignant tumor	3 (2.5)		
Undifferentiated malignant tumor	1 (0.8)		
Small cell carcinoma	1 (0.8)		
Leiomyosarcoma	1 (0.8)		
Mucinous adenocarcinoma	1 (0.8)		
Cystitis	1 (0.8)		
Squamous cell carcinoma	1 (0.8)		
Urothelial carcinoma	89 (74.8)		
Urothelial carcinoma + carcinoma in situ	19 (16.0)		

* Values are presented as number and percentage or as mean ± standard deviation and median (minimum–maximum), as appropriate. Pathological T stage was available for 118 patients; histological subtype was available for 119 patients. SD: standard deviation.

**Table 2:** Temporal changes in renal measurements of the right and left kidneys at baseline and 6, 12, and 24 months.

Measurements *	Preop Median (Min–Max)	6 Months Median (Min–Max)	12 Months Median (Min–Max)	24 Months Median (Min–Max)	*p*-Value
Right Kidney					
Renal pelvis AP diameter (mm)	6.4 (2.2–31.5)	6.2 (2.5–39.2)	6.4 (2.5–28)	6.3 (2.6–27.8)	0.038
Parenchymal thickness (mm)	18.1 (5.6–22.6)	17.3 (8.4–22)	17.5 (5.9–22.2)	17.5 (5.7–22.3)	<0.001
AP diameter/Parenchymal thickness	0.34 (0.13–5.03)	0.37 (0.15–2.22)	0.36 (0.15–2)	0.36 (0.15–4.28)	0.069
Kidney volume (mL)	175.1 (48.3–262.1)	169.3 (36.3–317)	168.8 (30.1–268.7)	165.9 (23.4–255.9)	<0.001
Left Kidney					
Renal pelvis AP diameter (mm)	6.9 (2.7–64)	7.4 (2.9–41)	7.5 (2.9–42)	7.1 (3.1–43)	0.064
Parenchymal thickness (mm)	17.6 (7.1–22)	16.7 (7–22.6)	16.9 (7–22)	16.6 (5.8–21.8)	<0.001
AP diameter/Parenchymal thickness	0.36 (0.16–9.0)	0.43 (0.14–5.86)	0.41 (0.14–6)	0.43 (0.15–6.23)	<0.001
Kidney volume (mL)	179.3 (82.8–292.5)	156.1 (51.8–395.7)	155.7 (63–289)	150 (47.7–300)	<0.001

* Values are presented as medians (minimum–maximum). *p*-values indicate differences across time points. AP: anteroposterior.

## Data Availability

The data presented in this study are available on request from the corresponding author. The data are not publicly available due to institutional privacy and ethical restrictions, as they are stored within the hospital’s secure electronic health record system.

## Supplementary Materials

The following supporting information can be downloaded at: https://www.mdpi.com/article/10.3390/jcm15135163/s1. Table S1. Baseline Demographic, Clinical, and Histopathological Comparison Between the Included Cohort and Patients Excluded Due to Incomplete Follow-up.

## Abbreviations

The following abbreviations are used in this manuscript:

RC	Radical cystectomy
MIBC	Muscle-invasive bladder cancer
RV	Renal volume
CT	Computerized tomography
AP	Anteroposterior
SFU	Society of Fetal Urology
eGFR	Estimated glomerular filtration rate
PDS	Polydioxanone
ROC	Receiver operating characteristic
SD	Standard deviation
RS	Rectosigmoid
