# Supplementary material for: Upper Urinary System Changes After Radical Cystectomy and Bricker Urinary Diversion: A Retrospective Evaluation of Functional and Radiological Parameters
Source: J Clin Med. 2026 Jul 2;15(13):5163. doi: 10.3390/jcm15135163 (PMC13362651; doi:10.3390/jcm15135163)
Supplement: Supplementary file 1 [file jcm-15-05163-s001.zip › jcm-4347786-supplementary.pdf]

Supplementary Table S1. Baseline Demographic, Clinical, and Histopathological Comparison Between the Included Cohort and Patients Excluded Due to Incomplete Follow-up.

Variables	Included Cohort (n=120)	Incomplete or Non-Standardized Longitudinal Follow-up (n=157)	p-value
**Age (years), Mean ± SD**	63±9	64.1±8.7	0.302[1]
**Sex**			0.614[2]
Male	100 (83.3)	128 (81.5)	
Female	20 (16.7)	29 (18.5)	
**Smoking**	101 (84.2)	135 (86.0)	0.675[2]
**Coronary artery disease**	33 (27.5)	40 (25.5)	0.709[2]
**Diabetes Mellitus**	36 (30.0)	44 (28.0)	0.722[2]
**Hypertension**	46 (38.3)	55 (35.0)	0.573[2]
**Preoperative Creatinine (mg/dL)**	1.08±0.4	1.11±0.35	0.504[1]
**Lymph node metastasis**	33 (27.7)	40 (25.5)	0.678[2]
**Positive surgical margin**	14 (11.8)	16 (10.2)	0.673[2]
**Neoadjuvant chemotherapy**	30 (25.0)	41 (26.1)	0.835[2]
**Adjuvant chemotherapy**	33 (27.5)	36 (22.9)	0.380[2]
**Pathological T stage**	118 (98.3)	157 (100)	0.758[2]
Organ-Confined(≤pT2/CIS/Ta/Tis)	70 (59.3)	96 (61.1)	
Advanced (≥pT3/pT4)	48 (40.7)	61 (38.9)	
**Histological subtype**	119 (99.1)	157 (100)	0.881[2]
Urothelial carcinoma	89 (74.8)	120 (76.4)	
Urothelial carcinoma + carcinoma in situ	19 (16.0)	22 (14.0)	
Non-urothelial variants/ Others	11 (9.2)	15 (9.6)	

* Values are presented as number and percentage or as mean±standard deviation , as appropriate. Pathological T stage was available for 118 patients; histological subtype was available for 119 patients in the included cohort. Pathological T stage distributions reflect the relative proportions available in the primary archival records. To ensure statistical validity and avoid low cell counts in categorical testing (Chi-square), T-stages were grouped into organ-confined vs. advanced disease, and histological subtypes were categorized as urothelial vs. non-urothelial variants.

All p-values > 0.05 indicate that no systematic selection or survivorship bias was introduced through logistical data attrition. [1]Independent t-test, [2]Chi-square test, SD: Standard Deviation
